# Relationship between self-esteem, self-efficacy and academic procrastination among medical students

**DOI:** 10.1192/j.eurpsy.2023.1169

**Published:** 2023-07-19

**Authors:** M. Turki, F. Sahnoun, A. Guermazi, O. Elleuch, F. Bennaceur, N. Halouani, S. Ellouze, J. Aloulou

**Affiliations:** Psychiatry B Department, Hedi Chaker University hospital, Sfax, Tunisia

## Abstract

**Introduction:**

Recent studies proved that academic procrastination is a very common pervasive problem that has a negative impact on general well-being, causing distress, anxiety, remorse and unhappiness. It could also result in poor academic performance and negatively affect students’ satisfaction with themselves and their academic life.

**Objectives:**

The aim of this study was to explore the influence of self-esteem and self-efficacy on academic procrastination among Tunisian medical students.

**Methods:**

We conducted a cross-sectional, descriptive and analytical study among medical students from Tunisia. Data were collected through an anonymous online questionnaire, exploring sociodemographic characteristics, the “Tuckman Procrastination Scale” (TPS), the “Rosenberg’s self-esteem scale” (RSES) and the “General Self-Efficacy Short Scale” (GSESS).

**Results:**

A total of 133 participants completed the questionnaire. Their mean age was 26 ± 3,8 years, with a sex-ratio (F/M) of 4,5. Among them 87.2% were engaged in academic procrastination, 57,1% showed low self-esteem and 55,6% perceived themselves as non-effective.

GSESS score were higher among males (p=0.019)

TPS score was negatively correlated with RSES score (p<0.001; r=-0.372). RSES score was positively correlated with GSESS score (p<0.001; r=0.44).

No relationship was proved between TPS and GSESS.

**Conclusions:**

Even though procrastination is most of the time considered as a maladaptive and detrimental behavior with a psychological cost, some authors consider it acting in a beneficial way, as it reflects self-reliance, autonomy and self-confidence knowing that they are able to finish their task in time. As a result, procrastination is linked to feelings of superiority and it should be recoined as “purposeful delay”.

**Disclosure of Interest:**

None Declared

